# Biodegradation of alkaline lignin by *Bacillus ligniniphilus* L1

**DOI:** 10.1186/s13068-017-0735-y

**Published:** 2017-02-21

**Authors:** Daochen Zhu, Peipei Zhang, Changxiao Xie, Weimin Zhang, Jianzhong Sun, Wei-Jun Qian, Bin Yang

**Affiliations:** 1 School of Environment and safty Engineering, Jiangsu University, Zhenjiang, Jiangsu China; 2 State Key Laboratory of Microbial Culture Collection and Application, Guangdong Institute of Microbiology, Guangzhou, Guangdong China; 3 Biological Sciences Division and Environmental Molecular Sciences Laboratory, Pacific Northwest National Laboratory, Richland, WA 99352 USA; 4 Bioproducts, Sciences and Engineering Laboratory, Department of Biological Systems Engineering, Washington State University, Richland, WA 99354 USA

**Keywords:** Alkaline lignin, *Bacillus ligniniphilus* L1, GC–MS, Proteomics

## Abstract

**Background:**

Lignin is the most abundant aromatic biopolymer in the biosphere and it comprises up to 30% of plant biomass. Although lignin is the most recalcitrant component of the plant cell wall, still there are microorganisms able to decompose it or degrade it. Fungi are recognized as the most widely used microbes for lignin degradation. However, bacteria have also been known to be able to utilize lignin as a carbon or energy source. *Bacillus ligniniphilus* L1 was selected in this study due to its capability to utilize alkaline lignin as a single carbon or energy source and its excellent ability to survive in extreme environments.

**Results:**

To investigate the aromatic metabolites of strain L1 decomposing alkaline lignin, GC–MS analysis was performed and fifteen single phenol ring aromatic compounds were identified. The dominant absorption peak included phenylacetic acid, 4-hydroxy-benzoicacid, and vanillic acid with the highest proportion of metabolites resulting in 42%. Comparison proteomic analysis was carried out for further study showed that approximately 1447 kinds of proteins were produced, 141 of which were at least twofold up-regulated with alkaline lignin as the single carbon source. The up-regulated proteins contents different categories in the biological functions of protein including lignin degradation, ABC transport system, environmental response factors, protein synthesis, assembly, etc.

**Conclusions:**

GC–MS analysis showed that alkaline lignin degradation of strain L1 produced 15 kinds of aromatic compounds. Comparison proteomic data and metabolic analysis showed that to ensure the degradation of lignin and growth of strain L1, multiple aspects of cells metabolism including transporter, environmental response factors, and protein synthesis were enhanced. Based on genome and proteomic analysis, at least four kinds of lignin degradation pathway might be present in strain L1, including a Gentisate pathway, the benzoic acid pathway and the β-ketoadipate pathway. The study provides an important basis for lignin degradation by bacteria.

**Electronic supplementary material:**

The online version of this article (doi:10.1186/s13068-017-0735-y) contains supplementary material, which is available to authorized users.

## Background

Lignin is a complex aromatic heteropolymer and is closely associated with cellulose and hemicellulose, which are the two major components of plant cell walls. Lignin is composed of phenylpropanoid aryl-C3 units linked together with a variety of ether and carbon–carbon linkages. While the structure of lignin has been extensively studied, it not yet been completely elucidated because of its complex and irregular matrix structure [1]. Currently, the lignocellulosic biofuel pretreatment process needs to remove or delocalize lignin, which might generate aromatic compounds that, as inhibitors, hinder enzymatic hydrolysis and fermentation [2]. Therefore, is an interesting strategy and a great challenge in the biofuels area to remove the negative effect of lignin in the process of saccharification, to biologically convert lignin into renewable liquid fuels and transfer to value-added products [3–5]. Lignin is very difficult to biologically degrade it because of its irregular matrix structure and lack of a standard, repeating covalent bond. So far, the degradation of lignin by microbes mostly focuses on fungi, especially white-rot basidiomycetes and related enzymes which include laccases, lignin peroxidase, and manganese peroxidase, all of which have been extensively studied [6–9]. The characteristic of lignin metabolism by bacteria is much less clarified compared with fungi, even though lignin degradation already have been found in several bacteria strains such as *Rhodococcus jostii*, *Streptomyces viridosporus* T7A, *Sphingobium* sp. SYK-6, *Nocardia, Pseudomonas, Comamonas*, *Bacillus*, and sulfate-reducing bacteria [10–16].

The currently identified bacteria capable of degrading lignin fall into three classes: actinomycetes, α-proteobacteria, and γ-proteobacteria, which were isolated from soil, sediments, animals, insect guts, etc. [15]. The pathways of lignin degradation were different in different bacteria, some bacteria contain oxidative enzymes to modify lignin by hydroxylation or demethylation, such as cytochrome P450 monooxygenases (P450s), dye-decolorizing peroxidase (DyP), laccase, manganese superoxide dismutase [17–19]. However, some bacteria such as *Rhodococcus jostii* RHA1 used the β-ketoadipate pathway (β-KAP) to degrade the lignin in the absence of hydrogen peroxide [20]. The β-KAP pathway is an enzyme-mediated aryl-ring degradation sequence, which converts aromatic compounds into metabolites of the tricarboxylic acid cycle (TCA) with nine essential enzymes and intermediates [21]. The β-KAP pathway including two branches, one branch converts protocatechuate by protocatechuate 3,4-dioxygenase, derived from phenolic compounds including p-cresol, 4-hydroxybenzoate, and numerous lignin monomers, to β-KAP. The other branch is catechol branch, which converts catechol, generated from various aromatic hydrocarbons, amino aromatics, and lignin monomers to β-KAP [22].

The goal of our study was to investigate the characteristics of alkaline lignin degradation by the alkaline bacteria *Bacillus ligniniphilus* L1. The GC–MS and intracellular comparison proteomic analysis were performed to explore metabolic of alkaline lignin in the intracellular of strain L1.

## Methods

### Strain and media

The strain used in this study is a halotolerant and alkaliphilic bacterium, *Bacillus ligniniphilus* L1 DSM 26145^T^, which was isolated from sediment from the South China Sea by our lab and deposited at the Japan Collection of Microorganisms (JCM 18543^T^) and German Collection of Microorganisms and Cell Cultures (DSM26145^T^) [11]. It is routinely cultured in marine 2216E medium (5 g tryptone, 1 g yeast extract, 34 g NaCl, and 0.1 g FePO_4_, 1000 ml sterile seawater). The mineral medium (MM63) used in this study as a limited carbon source medium was as follows: 100 mM KH_2_PO_4_, 75 mM KOH, 15 mM (NH_4_)_2_SO_4_, 1 mM MgSO_4_, 3.9 µM FeSO_4_, and 1 g (g/l) alkaline lignin (CAS8068-05-1, Catalog number 370959, Sigma–Aldrich, St. Louis. MO) or glucose as carbon source. In addition, in this research, lignin refers to alkaline lignin.

### Growth of *B. ligniniphilus* L1

Strain L1 was incubated in 2216E medium for 18 h at 30 °C, and the pellets were collected by centrifugation and washed with potassium phosphate buffer (100 mM) twice and re-suspended in MM63 medium. For growth culture, 1 ml of re-suspended cells was inoculated in 100 ml of MM63 medium with lignin or glucose as the carbon source and incubated in the shaker at 30 or 50 °C, 220 rpm. The glucose concentration in the culture following the growth of strain L1 was detected with SBA-40D Bio-sensor (Shandong Science Academic Biological Institute, PR China).

### Lignin degradation and decolorization analysis

The lignin concentration was determined by measuring sample absorbance at 280 nm as described before [23]. The color of the lignin culture was determined by the standard method of the Canadian Pulp and Paper Association [24, 25]. For decolorization analysis, 2 ml of culture was harvested by centrifugation at 12,000 rpm for 5 min to remove the cells, and 4 ml Na_2_HPO_4_–NaH_2_PO_4_ buffer (pH 7.6) was added to the supernatant. The absorbance of samples at 465 nm against distilled water was measured with a Beckman DU800 spectrophotometer (Beckman Coulter, Inc., Fullerton, CA). The absorbance values were transformed into color units (CU) according to the formula, CU = 500 × *A*_*2*_ */A*_*1*_, where *A*_*1*_ represents the A_456_ of a 500-CU platinum-cobalt standard solution (Cole-Parmer, USA) (0.132) and *A*_*2*_ is the absorbance of the sample. The decolorization proportion was defined as the ratio of the CU of the culture supernatant to that of the initial medium. All measurements were performed in triplicate.

For scanning electron microscopy analyses, the MM63 medium with 1 g lignin as the carbon source was inoculated with strain L1 and incubated for 7 days and with a sample uninoculated as control. Samples were centrifuged and the supernatant was freeze dried. Then the samples were mounted on aluminum stubs, coated with gold–palladium alloy, and examined with scanning electron microscopy (SEM, JSM-7001F, Japan).

### Gas chromatography–mass spectrometry (GC/MS) analysis

Bacterial samples (50 ml) were centrifuged (10,000-rpm for 15 min) to remove biomass. Then the supernatants were acidified to pH 2–3 with 6 M HCl and thoroughly extracted afterward with three volumes of ethyl acetate. The organic layer of the extraction mixture was collected and reduced to 10 ml by rotary evaporation at 37 °C and dewatered over anhydrous Na_2_SO_4_ to remove moisture. Then 100 µl of the organic layer was derivatized after the evaporation of the solvent under nitrogen stream. For the silylation procedure, 100 μl dioxane and 10 μl pyridine were added into samples and vortexed in glass tubes followed by silylation, which was performed with 50 μl of trimethyl silyl [*N*,*O*-bis(trimethylsilyl)trifluoroacetamide (BSTFA) and trimethylchlorosilane (TMCS), which were purchased from Sigma-Aldrich (St. Louis. MO)]. The mixture was heated in a water bath at 80 °C for 45 min with periodic shaking to dissolve the residues.

An aliquot of 1 ml of silylated mixture was injected into a GC–MS (AntoSystem XL GC-TurboMass; Perkin-Elmer, Waltham, MA, USA). The analytical column connected to the system was a PE-5MS capillary column (20 m × 0.18 mm internal diameter, 0.18 mm film thickness). The helium was used as a carrier gas with a flow rate of 1 ml/min. The column temperature program was 50 °C (5 min); 50–300 °C (10 °C/min, hold time: 5 min). The transfer line and the ion source temperatures were maintained at 200 and 250 °C. A solvent delay of 3.0 min was selected. In the full-scan mode, electron ionization mass spectra in the range of 30–550 (*m*/*z*) were recorded at electron energy of 70 eV. All standard monomeric phenolics compounds (1 mg) were derivatized and chromatographed as above. In order to identify the low molecular weight lignin-related compounds as trimethylsilyl (TMS) derivatives derived from bacterial treatment, their mass spectra were compared with that of the data of GC–MS spectral library (Wiley, NIST) available in the instrument and by comparing the retention time with those of some authentic compounds available.

### Proteomics

Cells were grown in MM63 medium with lignin as the single carbon source at 30 or 50 °C for 48 h, and then, 50 ml of culture were centrifuged to harvest the pellets for proteomics assays. For the extraction of protein, the pellets were suspended in a 20 mM sodium phosphate buffer, a pH 6.4, 1% protease inhibitor cocktail (Sigma-Aldrich, St. Louis, MO), 40 U/ml catalase (Sigma-Aldrich, St. Louis, MO), and 10 mM tributylphosphine (Applied Biosystems, CA, USA) maintained at 4 °C. The cells were mechanically disrupted with an Omni Ruptor 4000 Ultrasonic Homogenizer (Omni International, USA) by two 30 s cycles of homogenization at maximum speed, at 1 min intervals, and at 4 °C. Then, the suspension was centrifuged at 10,000 rpm for 20 min at 4 °C to remove the cell debris. To eliminate the cell envelope components, the supernatant was re-centrifuged at 22,000 rpm for 30 min at 4 °C. The protein concentration was determined by being in resuspension in 8 M urea and being assayed with Bicinchoninic acid (Thermo Scientific, Rockford, IL, USA). Proteomics analysis was performed as described before [26, 27]; the protein samples were determined using the isobaric tags for a relative and absolute quantification (iTRAQ) quantitative proteomic approach combined with a high-resolution, reversed-phase capillary liquid chromatography(LC) system coupled with a Thermo-Fisher Scientific LTQ-Orbitrap Velos mass spectrometer (San Jose, CA). The automated LC system was custom-built using two Agilent 1200 nanoflow pumps, one Agilent 1200 capillary pump (Agilent Technologies, Santa Clara, CA), and a PAL autosampler (Leap Technologies, Carrboro, NC). The liquid chromatography–tandem mass spectrometry (LC–MS/MS) raw data were converted into “.dta” files using Extract_MSn (version 3.0) from Bioworks Cluster 3.2 (Thermo Scientific). MS/MS spectra were identified based on database searching against the whole genome database of strain L1 (DDBJ/EMBL/GenBank. ANNK00000000) using the SEQUEST (version 27, revision 12) and MS-Align + algorithms [28]. The cluster analysis of detected proteins was performed using the GoMiner and GenMAPP programs.

## Results and discussion

### Growth of strain L1 on the lignin medium

**Fig. 1:**
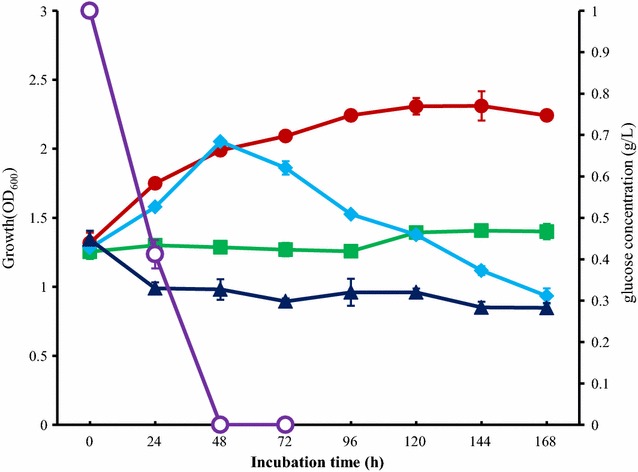
Growth of strain L1 during 7 days incubation with lignin or glucose as carbon source. Symbols: *closed circles* glucose and lignin as carbon source incubated at 50 °C; *closed squares* lignin as single carbon source incubated at 50 °C; *closed diamond* glucose and lignin as carbon source incubated at 30 °C; *closed triangles* lignin as single carbon source incubated at 30 °C; *open circles* the concentration of glucose during incubation of strain L1 in culture with glucose and lignin as carbon source

To investigate the growth of strain L1 with lignin or glucose as the carbon source, cells were incubated in MM63 medium with lignin and glucose or only lignin as the carbon source at 30 °C or 50 °C for 7 days with pH 9. The growth curve showed that strain L1 is able to slightly grow at 50 °C but not at 30 °C with lignin as the single carbon source (Fig. 1). It indicates that lignin is able to support the growth of strain L1 as the carbon source at higher incubation temperatures, but not at 30 °C, the optimal growth temperature of strain L1. In addition, when the incubation temperature increased from 30 to 50 °C, the stationary phase was extended from 72 to 144 h in the batch incubation with glucose and lignin as the carbon source. This is because when glucose was exhausted after 48 h incubation (Fig. 1), the lignin provided energy as carbon source to support the growth of strain L1 at 50 °C. These results suggested that the incubation temperature at 50 °C was an optimal option for the degradation of lignin by strain L1. Therefore, we selected at 50 °C for the incubation of strain L1 in the following section. Some papers report that fungi can break down lignin but the glucose had to be added as an energy source in order for lignin to be digested. Digesting lignin without adding glucose was a failure in *Polyporus versicolor* and two other wood-rotting fungi [29, 30]. For some bacteria, another carbon source is also necessary for lignin degradation. One example of this is *Enterobacter lignolyticus* SCF1, which requires the addition of xylose [31]. Despite the fact that the complete oxidation of lignin is highly exothermic, the microbial degradation of lignin actually needs an energy source, and lignin degradation is too slow to serve as a source of metabolic energy. This might be the reason why many microorganisms cannot utilize lignin as their single carbon or energy source. However, strain L1 is tolerant at high temperatures (more than 50 °C), and higher incubation temperatures might lead to lignin to be more easily degraded by strain L1.

### Lignin degradation by strain L1

**Fig. 2:**
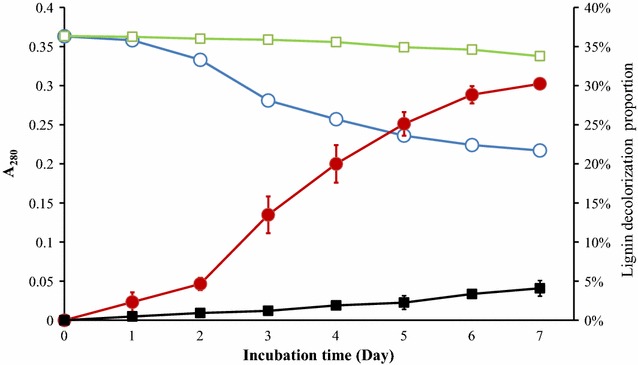
Lignin degradation and decolorization rate during 7 day’s incubation. Symbols: *open circle* lignin concentration; *open squares* uninoculated sample’s lignin concentration; *closed circle* lignin decolorization proportion; *closed squares* uninoculated sample’s lignin decolorization proportion

**Fig. 3:**
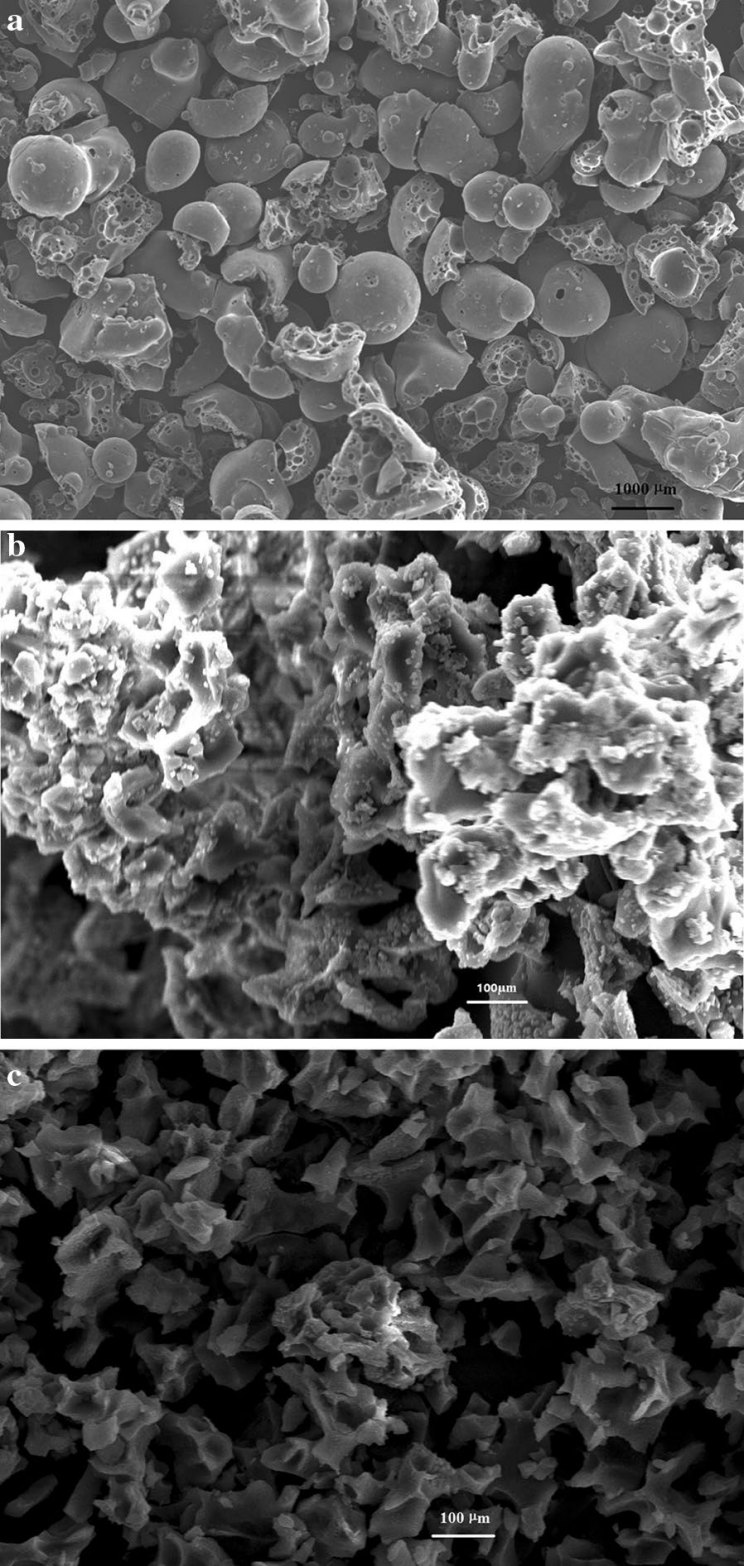
Scanning electron micrograph of lignin. **a** Untreated lignin, **b** lignin treated by 7 days incubation in MM63 medium with lignin as single carbon source without strain, **c** lignin treated by 7 days degradation with strain L1 in MM63 medium with lignin as single carbon source

To determine the degradation rate of lignin by strain L1, the absorbance at A_280_ was detected every 24 h during 7 days of incubation with lignin as the sole carbon source in MM63 medium. Results showed that the A_280_ was reduced from 0.36 to 0.22 during the 7-day incubation (Fig. 2). This means that about 38.9% of the lignin was degraded by strain L1 during the 7 days of incubation. In addition, the morphologies of the untreated lignin and treated by strain L1 are investigated by SEM and shown in Fig. 3. The untreated lignin consists of small balls, ovals, or spherical fragments, while treated lignin by 7 days incubation with or without strain L1 both became irregular fragments. However, the particle size and morphology of lignin treated by strain L1 was different with that of without strain L1 in culture. The lignin treated in culture without strain L1 showed bigger particle size and have many tiny particles adhesions in surface. Most of the fragments of the untreated lignin were within the size range 200–3000 µm and treated in cultures without strain during 7 days incubation, the size range reduced to 150–500 µm. When degraded by strain L1, the fragment size range was reduced to 30–300 µm. The variation of size and shape suggested that the lignin was degraded by strain L1. To detect the color removal of the lignin medium by strain L1, cells were incubated for 7 days in MM63 medium with 1 g lignin at pH 9 at 50 °C. The time course for the decolorization rate of the strain L1 culture is shown in Fig. 2. The maximum color removal reached 30% at the 7th day. The decolorization of lignin also confirmed that lignin actually degraded because of strain L1. Previous research has shown that manganese peroxidase (MnP) and laccase are thought to have an important role in the decolorization of lignin [24]. It is suggested that strain L1 should able to secrete MnP or laccase for lignin degradation.

### GC–MS spectrometry analysis

To identify compounds in the complex matrix, GC–MS was a highly specific method, was able to provide a confirmatory identification result, and has already been used to analyze compounds with low molecular weight from the degraded lignin product [32, 33]. To observe the variation of residual lignin during the lignin degradation process of strain L1, cells were incubated in MM63 medium for 7 days at a temperature of 50 °C with lignin as the sole carbon source and the fractions collected from culture were detected every 24 h.

The aromatic compounds identified from the peaks of the chromatogram of the uninoculated (control) and the inoculated cultures with L1 are shown in Table 1. In total, 15 aromatic compounds were identified from L1 cultures during the 7 days of incubation, and only 9 aromatic compounds were observed in the control sample.

**Table 1:** Dimensions of degradation products from lignin

No.	Retention time	Compounds		Control
1	13.644	Guaiacol	C_7_H_8_O_2_	–
2	13.917	Benzoic acid	C_7_H_6_O_2_	+
3	14.566	Benzene, 1-methyl-2-(1-methyl-2-propenyl)-	C_11_H_14_	–
4	14.613	Benzaldehyde, 3,4-dimethyl-	C_9_H_10_O	–
5	14.82	Phenylacetic acid	C_8_H_8_O_2_	+
6	16.192	4-Hydroxybenzaldehyde	C_7_H_6_O_2_	+
7	18.496	4-Hydroxy-benzoic acid	C_7_H_6_O_3_	+
8	18.562	Vanillin	C_8_H_8_O_3_	+
9	18.835	4-hydroxyphenylacetic acid	C_8_H_8_O_3_	–
10	19.549	4-Hydroxyacetophenone	C_8_H_8_O_2_	+
11	20.339	Vanillic acid	C_8_H_8_O_4_	+
12	20.659	4-Allyl-2-methoxyphenol	C_10_H_12_O_2_	+
13	20.725	4-Hydroxy-3,5-dimethoxybenzaldehyde	C_9_H_10_O_4_	–
14	21.797	4-(3-Hydroxybutyl)-2-methoxyphenol	C_11_H_16_O_3_	–
15	22.521	(3-Ethoxy-4-hydroxyphenyl) (hydroxy) acetic acid	C_10_H_12_O_5_	–

“–”, compound was not detected; “+”, compound was detected; all compounds were detected in cultures

**Fig. 4:**
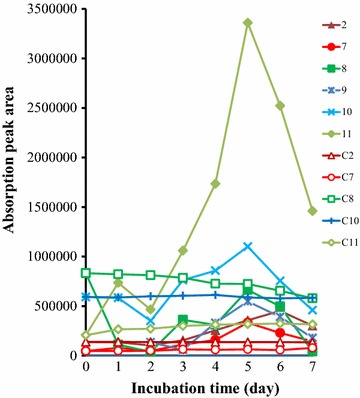
The variation of absorption peaks area of part aromatic compounds identified by GC–MS during 7 days’ incubation with lignin as single carbon source with uninoculated sample as control. *Numbers* represent the aromatic compounds were same with Table 1, and *C2*, *C7*, *C8*, *C9*, *C10,* and *C11* represent the compounds peaks of control sample

**Fig. 5:**
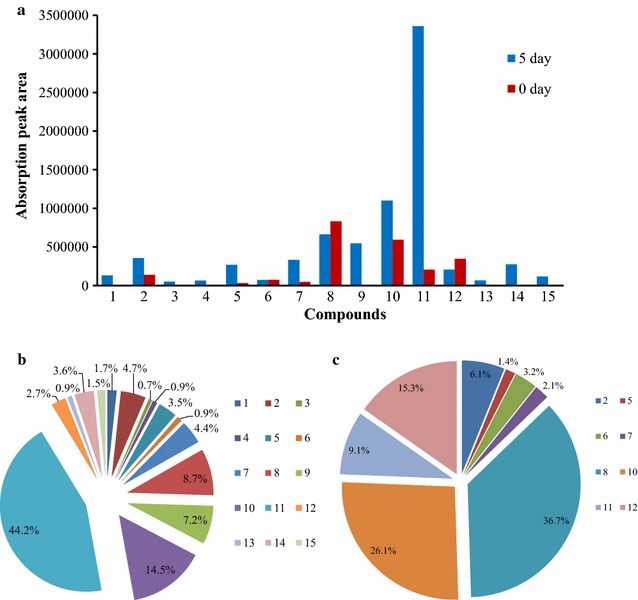
Comparison of absorption peaks of aromatic compounds identified by GC–MS during culturing process. **a** The variation of absorption peaks at first and fifth days’ incubation. **b** The proportion of absorption peaks area of aromatic compounds in the fifth days’ incubation. **c** The proportion of absorption peaks area of aromatic compounds in the control samples. *Numbers* represent the aromatic compounds were same with Table 1

Most of the absorption peak area of the aromatic compounds increased during the 7 days of incubation and it reached its highest point during the fifth day (Fig. 4). The peaks of control (uninoculated sample) during 7 days incubation showed no obvious fluctuation except C8 (vanillin), which decreased following the incubation process, it might because vanillin is unstable in the alkaline culture and was oxidized during 7 days incubation. The increasing of the peak’s area means that the concentration of compounds increased during the 7 days of incubation. Among the aromatic metabolites of lignin degradation, vanillic acid, phenylacetic acid, and 4-hydroxy-benzoicacid have higher absorption peak area variation, which increased 16, 8, and sevenfold, respectively, during the 7-day incubation. However, the concentration of 4-allyl-2-methoxyphenol (eugenol) and the vanillin concentration decreased following the sustained incubation (Fig. 5a).

Vanillic acid (compound 11) was the most abundant aromatic metabolite of lignin based on the calculation of the peak area of the fifth day’s chromatogram and constituted 44.2% of all aromatic metabolites produced. Other predominate aromatic compounds included 4′-hydroxyacetophenone (compound 10) (14.5%), vanillin (compound 8) (8.7%), and 4-hydroxyphenylacetic acid (compound 9) (7.2%) (Fig. 5b). Vanillin also took up the largest proportion of the uninoculated sample and resulted in being 36.7% of the sample; other predominate aromatic compounds including 4′-hydroxyacetophenone (26.1%), 4-allyl-2-methoxyphenol (compound 12) (15.3%), vanillic acid (9.1%), and benzoic acid (compound 2) (6.1%) were observed (Fig. 5c). It suggested that not only lignin was degraded by strain L1 but also that the aromatic compounds from lignin also were degraded or utilized by strain L1 as a carbon or energy source. Vanillic acid and vanillin as the predominant compounds of degraded lignin have already been identified in previous reports [34–37]. In that report, the depolymerization and oxidation of lignin produced compounds predominantly including vanillic acid, benzoic acid, 1,2-benzenedicarboxylic acid, vanillin, vanillic acid, 4-hydroxy-benzoic acid, and 4-hydroxy-3,5-dimethoxybenzaldehyde, which were all identified with GC–MS from hydrolytically degraded biomass [37].

Lignin is the most abundant aromatic substance in the biosphere, and the exact structure of protolignin, the untreated lignin found in plants, is still unknown. Based on a review of Joseph Zakzeski et al., more than 160 kinds of single phenyl ring compounds are able to be obtained from lignin, including β-*O*-4, 5-5, β-1, α-1, α-*O*-4, and 4-*O*-5 bond linkage model compounds, which resemble *p*-coumaryl alcohol and its derivatives, coniferyl alcohol, which resembles lignin model compounds and its derivatives, and sinapyl alcohol, which also resembles lignin model compounds and its derivatives [1]. There are fifteen single phenyl ring aromatic compounds that were identified from residual lignin degraded by L1, and there might be many more aromatic compounds from residual lignin that might have been unable to be identified by GC–MS because of their presence in small amounts which might be out of the measure range of GC–MS.

### Comparison of the proteome profiles of L1 from Lignin or glucose as carbon source

Proteomic studies showed the great potential for studying in large-scale metabolism and are able to help for the activation of various biosynthetic pathways of bacteria, which can be observed in the corresponding media [38]. Comparative proteomic analyses were performed to determine the variation of intracellular proteins, with glucose or lignin as the single carbon source. Approximately 1447 kinds of protein were produced by proteomic analysis. In total, 324 proteins showed a significantly different abundance and 141 proteins were at least twofold up-regulated in the presence of lignin. In addition, when cells were grown in the presence of lignin at 30 or 50 °C, there were 618 proteins that were significantly abundant and 500 proteins that were at least twofold up-regulated at 50 °C (Table 2). The functions of the corresponding proteins were assigned based on the Kyoto Encyclopedia of Genes and Genomes (KEGG) database (http://www.genome.jp/kegg/). As shown in Additional files 1 and 2, most of the differently expressed proteins fell into the following categories: (i) ATP binding cassette (ABC) transport system; (ii) protein synthesis and assembly; (iii) lignin degradation; (iv) environmental response factors; (v) amino acid metabolism; (vi) RNA and DNA metabolism; (vii) pyrimidine metabolism; (viii) the citrate cycle; (ix) flagellar assembly; (x) fatty acid metabolism; (xi) phosphoenolpyruvate-carbohydrate phosphotransferase system (PTS); (xii) ribosomes; (xiii) sporulation, and other pathways.

**Table 2:** Proteomic data for differential regulation in lignin compare to glucose and temperature 50–30 °C

	Up-regulated	Down-regulated
Lignin	141	183
Temperature	500	118

The up-regulated proteins which might be related with lignin degradation, including peroxiredoxin, cytochrome oxidase, oxidoreductase, ferredoxin, aminodeoxychorismate lyase, dehydrogenase, acetyl-CoA C-acetyltransferase, and enoyl-CoA hydratase etc. In the lignin degradative process, hydrogen peroxide oxidation catalyzed by ligninolytic peroxidases was identified as a key reaction [39]. Cytochrome P450 peroxidase, as mixed function oxidases, belongs to a superfamily of heme-thiolate proteins that can catalyze a variety of enzymatic reactions to transform xenobiotic chemicals into more polar and/or detoxified derivatives, and as an oxidase, involved in the lignin degradation [6, 40–42]. Cytochrome P450-related gene was not observed but many present cytochrome c551 and cytochrome c and b related genes in the whole genome of strain L1 and cytochrome c551 and the cytochrome c protein were up-regulated in strain L1. Therefore, cytochrome c551 and cytochrome c peroxidase might play an important role in the lignin degradative process. 2-cys peroxiredoxins (Prx) have 2.6-fold of expression with lignin as substrate was observed. Prx are a family of cysteine-dependent peroxidases that react with hydrogen peroxide, aliphatic and aromatic hydroperoxide substrates, and peroxynitrite [43]. In bacteria, Prx are critical components of the antioxidant defense systems and regulate a variety of signaling processes including reactive oxygen species scavenging, cell proliferation, differentiation, and cell death [44, 45]. Reactive oxygen species are proven to be able to mediate the expression of lignin peroxidase. Therefore, the over-expression of 2-cys peroxiredoxin might have improved the degradation of lignin in strain L1. Other up-regulated protein involved in lignin degradation such as short chain dehydrogenase (twofold), carbon-monoxide dehydrogenase (fivefold), acetyl-CoA acetyltransferase (fourfold), enoyl-CoA hydratase (twofold), ferredoxin (twofold), and formate dehydrogenase (twofold) were observed. Three short chain dehydrogenase were identified which involve the catabolism of arylglycerol-β-aryl ether, which is an important model compound of lignin [46]. Acetyl-CoA acetyltransferase and enoyl-CoA hydratase are involved in the degradation of the aromatic compound benzoate, as described before [47]. Ferredoxin reductase and ferredoxin play a role in lignin and aromatic acid degradation pathways in the bacterium [48, 49]. Formate dehydrogenase is involved in oxalate metabolism, as the presence of oxalate is able to improve the degradation of lignin by stimulating the Mn peroxidase activity in its ability to chelate Mn^2+^ [50]. Quinone oxidoreductase has 2.6-fold of expression, which are involved in lignin oxidation [51].

Environmental response factors such as the general stress protein, the repressor LexA, the DNA integrity scanning protein, the catabolite repression HPr-like protein, the central glycolytic genes regulator, the transcriptional regulator, the rod shape-determining protein, and the methyl-accepting chemotaxis protein were up-regulated. Many aromatic compounds from lignin were toxic to bacteria cells, such as the lignin degradation products benzoic acid and β-coumaric acid, which were both toxic to *Cupriavidus necator* cells [52]. The stress of the toxins in the cells of L1 might induce the up-regulation of environmental response factors. Environmental response factors such as repressor LexA, the central glycolytic genes regulator, and the general stress protein were up-regulated. The transcriptional repressor LexA is a key component of the SOS response, which is an important gene for the regulation of DNA repair and is crucial for bacterial survival [53]. The methyl-accepting chemotaxis protein and the methyl-accepting chemotaxis sensory transducer have been significantly up-regulated when lignin was the single carbon source and resulted in 5.5- and 10-fold of expression. Methyl-accepting chemotaxis proteins already been proved to be involved in triggering chemotaxis towards aromatic compounds such as benzoate, vanillic acid, vanillin, catecho, fumarate, protocatechuate, and phenol, which is important for the degradation of aromatic compounds [54]. The purpose of bacterial chemotaxis is to control the direction of flagellar rotation [55]. Therefore, the up-regulation of flagellar assembly proteins might contribute to the degradation of aromatic compounds.

When lignin was used as the single carbon source, most of the proteins related with transporter systems were up-regulated. Twenty-three kinds of protein were up-regulated, but only 2 proteins were down-regulated. The ABC transporters are central to many physiological processes, including the uptake of nutrients, the non-classical secretion of signaling molecules and toxins, which are able to transport an enormous variety of substrates, ranging from small ions to large proteins. They can also act as mediators and regulators in transmembrane signaling processes, perhaps without mediating any direct transport reaction. The degradation of lignin by strain L1 is a complicated process and involves many enzymes and regulation factors. We speculate that the up-regulation of the ABC transporter system might improve the transport ability of enzymes and related regulation factors as well as the intake of aromatic compounds for lignin degradation (Additional file 1). Crystalline bacterial cell surface layer (S-layer) proteins that have 2.7-fold of expression with lignin as the single carbon source were observed (Additional file 1). S-layer proteins are formed by the self-assembly of monomeric proteins into a regularly spaced, two-dimensional array and have important roles in growth and survival. Their many functions include the maintenance of cell integrity, enzyme display, and the provision of adhesion sites for exoproteins [56]. Therefore, the S-layer proteins might have an important role in the degradation of lignin by providing adhesion sites for enzymes secreted by strain L1, it needs classic genetics experiments to confirm this speculation in future work. ATP synthase subunit alpha, beta, and gamma proteins have 2.2-, 2.1-, and 2.0-fold of expression with lignin as the sole carbon source. The up-regulation of ATP synthase also was observed in *Enterobacter lignolyticus* SCF1 during lignin degradation [31]. It might be that the lignin, as a substrate, requires more energy to decompose because of its complicated matrix ring structure.

Using lignin as the single carbon source severely repressed the expression of ribosomal proteins, such as 50S ribosomal proteins L2, L4, L6, L13, L14, L16, L18, L20, L22, L23, L25, and L27, while 30S ribosomal proteins S7, S15, S18, and S19 were observably down-regulated. Nutrition starvation induced the bacterial stringent response trigged by ribosomes, which serve as the sites of biological protein synthesis and cell growth. Therefore, the down-regulation of ribosomal protein would inhibit the growth of cells and would make the cells secrete more enzymes for the degradation of lignin. The citrate cycle pathway was down-regulated and 8 proteins that were more than twofold were expressed. The down-regulation of citrate cycle proteins which might be using the lignin as a carbon source is not as efficient as glucose to provide energy for growth of cells. In addition, aminoacyl tRNA synthetase was also down-regulated. It plays an important role in DNA translation, specifically, the expression of genes to create proteins from the ribosomes. Interestingly, the unique phenomenon was observed that 8 kinds of sporulation-related proteins were down-regulated. It is still unclear why using lignin as the substrate influenced the sporulation of bacteria.

In summary, during the progression of lignin degradation by strain L1, the intracellular secretory capacity of cells tend to upregulate key proteins related with utilizing lignin as the single carbon source, including enzymes involving lignin degradation, transport system, environmental response factors, and energy metabolism.

### Lignin degradation pathway of strain L1 putatively based on whole genome and GC–MS

**Fig. 6:**
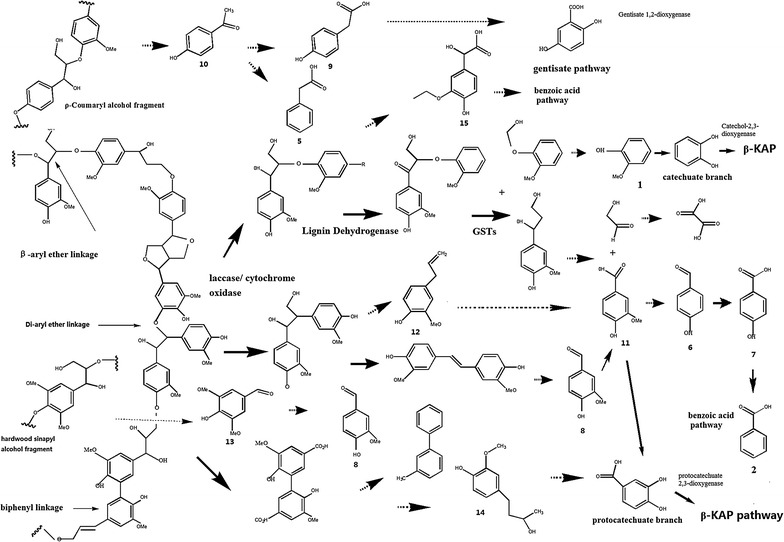
Putative lignin degradation pathways of strain L1. *Numbers* represent the aromatic compounds were same with Table 1

The whole genome sequence of strain L1, already obtained by the Illumina/Solexa HiSeq2000 sequencing system, was based on the genome data and the previous literature about enzymes involving in lignin degradation (Whole Genome Shotgun project has been deposited at DDBJ/EMBL/GenBank under the accession no. ANNK00000000). We summarized the genes which might have been involved in the lignin degradation in Table 3. Two multicopper oxidase gene sequences were observed in the genome of L1, which might be laccase genes. There are many multicopper oxidase genes that already were identified from genus *Bacillus* with laccase activity [57–59]. The decolorization results also suggest that strain L1 is might able to secret laccase. However, no MnP or LiP gene was observed in whole genome of strain L1. Based on the GC–MS and genome data, there might be 3 pathways present for lignin degradation in strain L1: the gentisate pathway, the benzoic acid pathway, and the β-ketoadipate pathway. In addition, the β-ketoadipate pathway includes the catechuate branch and the protocatechuate branch (Fig. 6). Three gentisate 1, 2-dioxygenase genes were observed in the whole genome of L1, and which indicated the presence of the gentisate pathway in strain L1. In the benzoic acid pathway, 4-hydroxybenzoate decarboxylase (whole genome of L1) catalyzes the 4-hydroxy-benzoic acid (compound 7) to benzoic acid (compound 2), and benzoic acid converted into cis-diols by toluate 1, 2-dioxygenase (one toluate 1, 2-dioxygenase gene was observed in the genome of L1) [60]. For catechuate branch, guaiacol (compound 1) converts to catechol by demethylation, and catechol was open ring by catechol 2, 3-dioxygenase (two genes were observed in the whole genome of L1). In the protocatechuate branch, 4-hydroxybenzaldehyde (compound 6) was converted into 3, 4-dihydroxybenzoate (protocatechuate) by 4-hydroxybenzoate 3-monooxygenase (one 4-hydroxybenzoate 3-monooxygenase gene was identified in the whole genome of L1), and protocatechuate was catalyzed by protocatechuate 2, 3-dioxygenase (one protocatechuate 2, 3-dioxygenase gene was identified in the whole genome of L1) [61].

**Table 3:** Putative gene involving lignin degradation of L1

Orf_name	Length (bp)	Encoding protein
gm_orf2082	3693	Multicopper oxidase
gm_orf357	1515	Multicopper oxidase
gm_orf140	750	Heme peroxidase
gm_orf845	501	Thioredoxin peroxidase
gm_orf2135	2058	Catalase-peroxidase
gm_orf545	195	Glycolate oxidase
gm_orf546	609	Glycolate oxidase
gm_orf548	108	Glycolate oxidase
gm_orf549	1071	Glycolate oxidase
gm_orf547	633	Glycolate oxidase
gm_orf2461	894	Superoxide dismutase
gm_orf2691	609	Superoxide dismutase
gm_orf3186	960	Catechol 2,3-dioxygenase
gm_orf726	852	Catechol 2,3-dioxygenase
gm_orf2069	981	Catechol 2,3-dioxygenase
gm_orf1879	822	Protocatechuate 2,3-dioxygenase
gm_orf1866	525	Biphenyl 2,3-dioxygenase
gm_orf1846	582	4-Hydroxybenzoate decarboxylase
gm_orf2090	582	4-Hydroxybenzoate decarboxylase
gm_orf2161	1035	Phenol hydroxylase
gm_orf2068	1215	Phenol hydroxylase
gm_orf2065	483	Phenol hydroxylase small subunit
gm_orf3648	1101	Aryl-alcohol dehydrogenase
gm_orf3660	1098	Aryl-alcohol dehydrogenase
gm_orf3713	1146	3-Hydroxybenzoate 6-monooxygenase
gm_orf129	1437	4-Hydroxyphenylacetate 3-monooxygenase
gm_orf1870	1215	4-Hydroxybenzoate 3-monooxygenase
gm_orf1914	1149	S-(hydroxymethyl)glutathione dehydrogenase
gm_orf3933	1137	S-(hydroxymethyl)glutathione dehydrogenase
gm_orf100	402	Glutathione transferase
gm_orf1871	933	5-Carboxy-2-hydroxymuconate-6-semialdehyde decarboxylase
gm_orf1873	1470	2-Hydroxymuconic semialdehyde dehydrogenase
gm_orf3707	1434	5-Carboxymethyl-2-hydroxymuconate delta-isomerase
gm_orf3527	885	5-Carboxymethyl-2-hydroxymuconate delta-isomerase
gm_orf1956	489	Salicylate 1-hydroxylase beta subunit
gm_orf3187	1578	4-Hydroxyphenylacetate 3-hydroxylase
gm_orf2162	1104	Methane/phenol/toluene monooxygenase
gm_orf3714	1113	Gentisate 1,2-dioxygenase
gm_orf3703	1119	Gentisate 1,2-dioxygenase
gm_orf1830	537	Gentisate 1,2-dioxygenase
gm_orf3117	1257	Phthalate 4,5-dioxygenase oxygenase subunit
gm_orf2759	1257	Phthalate 4,5-dioxygenase oxygenase subunit
gm_orf1962	1287	Phthalate 4,5-dioxygenase
gm_orf1829	1284	Phthalate 4,5-dioxygenase
gm_orf2094	1335	3-Chlorobenzoate-3,4-dioxygenase oxygenase subunit
gm_orf1964	984	Putative ring-cleaving dioxygenase mhqA
gm_orf1957	1248	Terephthalate 1,2-dioxygenase oxygenase large subunit
gm_orf1865	1353	Polycyclic aromatic hydrocarbon dioxygenase large subunit
gm_orf1409	552	2-Cys peroxiredoxin
gm_orf2729	369	Cytochrome c551
gm_orf521	408	Cytochrome c551
gm_orf1456	1827	K02274 cytochrome c oxidase subunit I
gm_orf1455	1041	Cytochrome c oxidase subunit II
gm_orf2949	1155	Aminodeoxychorismate lyase
gm_orf2519	744	Short chain dehydrogenase/reductase family oxidoreductase
gm_orf2443	1656	Cytochrome c biogenesis protein
gm_orf3584	486	Carbon-monoxide dehydrogenase small subunit
gm_orf178	363	Acetyl-CoA C-acetyltransferase
gm_orf2189	777	1489 enoyl-CoA hydratase/isomerase
gm_orf2421	294	Ferredoxin
gm_orf1543	840	Formate dehydrogenase accessory protein
gm_orf2481	1017	Luciferase-type oxidoreductase
gm_orf1315	1422	Dihydrolipoamide dehydrogenase
gm_orf59	1500	Malate dehydrogenase (quinone)

## Conclusions

Based on above data and analysis, we conclude that *Bacillus ligniniphilus* L1^T^ able to degrade alkaline lignin and 15 aromatic compounds were identified during the degradation process of lignin. There present 3 pathways for lignin degradation in strain L1, including gentisate pathway, benzoic acid pathway, and the β-ketoadipate pathway, and β-ketoadipate pathway including catechuate and protocatechuate two branch. Overall, this study is an attempt to investigate the aromatic metabolites of lignin used by strain L1, and analyze the variation of the intracellular protein expressed by proteomic analysis to explain the physiological phenomena of strain L1 with lignin as its only carbon source. To completely understand the metabolic characteristics of lignin and identify the enzymes involved in the decomposition process by strain L1, more experiments and approaches such as systems biology study are needed in future work.

## Additional files

**Additional file 1.** Up regulation of expressed protein with lignin as carbon source.

**Additional file 2.** Down regulation of expressed protein with lignin as carbon source.

## Abbreviations

GC–MS: gas chromatography–mass spectrometry
P450s: cytochrome P450 monooxygenases
MM63: mineral medium
OD: optical density
CU: color units
SEM: scanning electron microscopy
BSTFA: *N*,*O*-bis(trimethylsilyl)trifluoroacetamide
TMCS: trimethylchlorosilane
iTRAQ: isobaric tags for a relative and absolute quantification
TMS: trimethylsilyl
LC–MS/MS: liquid chromatography–tandem mass spectrometry
ABC: ATP binding cassette
PTS: phosphoenolpyruvate-carbohydrate phosphotransferase system
Prx: 2-cys peroxiredoxin
S-layer: surface layer
